# Initial Experience with a Wireless Ultrasound-Guided Vacuum-Assisted Breast Biopsy Device

**DOI:** 10.1371/journal.pone.0144046

**Published:** 2015-12-02

**Authors:** E-Ryung Choi, Boo-Kyung Han, Eun Sook Ko, Eun Young Ko, Ji Soo Choi, Eun Yoon Cho, Seok Jin Nam

**Affiliations:** 1 Department of Radiology and Center for Imaging Science, Samsung Medical Center, Sungkyunkwan University School of Medicine, Seoul, Korea; 2 Department of Pathology, Samsung Medical Center, Sungkyunkwan University School of Medicine, Seoul, Korea; 3 Department of General Surgery, Samsung Medical Center, Sungkyunkwan University School of Medicine, Seoul, Korea; School of Medicine, Fu Jen Catholic University, TAIWAN

## Abstract

**Objective:**

To determine the imaging characteristic of frequent target lesions of wireless ultrasound (US)-guided, vacuum-assisted breast biopsy (Wi-UVAB) and to evaluate diagnostic yield, accuracy and complication of the device in indeterminate breast lesions.

**Materials and Methods:**

From March 2013 to October 2014, 114 women (age range, 29–76 years; mean age, 50.0 years) underwent Wi-UVAB using a 13-gauge needle (Mammotome Elite^®^; Devicor Medical Products, Cincinnati, OH, USA). In 103 lesions of 96 women with surgical (n = 81) or follow-up (n = 22) data, complications, biopsy procedure, imaging findings of biopsy targets and histologic results were reviewed.

**Results:**

Mean number of biopsy cores was 10 (range 4–25). Nine patients developed moderate bleeding. All lesions were suspicious on US, and included non-mass lesions (67.0%) and mass lesions (33.0%). Visible calcifications on US were evident in 57.3% of the target lesions. Most of the lesions (93.2%) were nonpalpable. Sixty-six (64.1%) were malignant [ductal carcinoma *in situ* (DCIS) rate, 61%] and 12 were high-risk lesions (11.7%). Histologic underestimation was identified in 11 of 40 (27.5%). DCIS cases and in 3 of 9 (33.3%) high-risk lesions necessitating surgery. There was no false-negative case.

**Conclusion:**

Wi-UVAB is very handy and advantageous for US-unapparent non-mass lesions to diagnose DCIS, especially for calcification cases. Histologic underestimation is unavoidable; still, Wi-UVAB is safe and accurate to diagnose a malignancy.

## Introduction

Percutaneous breast biopsy became an alternative to surgical biopsy for imaging-detected or palpable breast masses. Accordingly, the needle biopsy has evolved from the Tru-Cut biopsy technique to vacuum-assisted breast biopsy (VAB), with the goal of minimizing a wrong diagnosis. VAB provides larger tissue samples with consistent quality. The advantages of VAB have been reported as a lower mistargeting rate and a lower underestimation rate of cancer, compared with a Tru-Cut biopsy using a 14-gauge needle [1–4].

However, conventional VAB with an 8- to 14-gauge needle is cumbersome due to the large equipment including a connecting line and the requirement for more time to prepare this equipment. Additionally, the cost of conventional VAB is much higher than for the Tru-Cut biopsy.

To overcome these disadvantages, a new wireless ultrasound (US)-guided VAB device (Wi-UVAB) has been introduced. The Mammotome Elite^®^ (Devicor Medical Products, Cincinnati, OH, USA) automatically transports the acquired tissue into a single chamber. The device is easy to handle because of the lack of a connecting line and no additional time is required to prepare the equipment.

To our knowledge, only one study has evaluated the usefulness of this new Wi-UVAB device since its introduction. ^5^ The diagnostic accuracy and success rate are unclear. The purpose of this study was to determine the imaging characteristics of frequent target lesions of Wi-UVAB and to evaluate diagnostic yield, accuracy and device-related complications in breast lesions.

## Materials and Methods

Between March 2013 and December 2014, 114 consecutive female subjects (age range, 29–76 years; mean age, 50.0 years) underwent Wi-UVAB for 122 breast lesions using Mammotome Elite^®^ with a 13-gauge needle. We retrospectively reviewed the medical records and imaging findings to determine the biopsy indications, complications and core and final pathological diagnosis. Eighteen patients with 19 lesions were excluded as follow-up US was not available or less than 6 months. The final study population consisted of 103 lesions in 96 women.

Our institutional (Samsung Medical Center [SMC]) review board approved our study (SMC 2014-07-118-001) with a waiver of the requirement for informed consent.

### Biopsy procedure

Because our hospital is a tertiary referred hospital, our breast clinic is enriched by complicated cases including cases with imaging-pathologic discordance after the first biopsy and cases deemed to be difficult in US-guided Tru-Cut biopsy. After the review of outside US images and targeted US for suspected area, we recommended Wi-UVAB in the lesions that were seemingly difficult to target with Tru-Cut gun biopsy.

All biopsies were guided using a model IU 22 high-resolution US machine equipped with a 5- to 12-MHz linear transducer (Philips Medical System, Bothell, WA, USA). The biopsies were performed by one of four radiologists with 4 to 17 years of experience in breast sonography and intervention. The patients lay in the supine or oblique supine position. After visualization of the lesion using US, the skin entrance was determined for needle advancement parallel to the US probe. Local anesthesia was administered before the biopsy from the skin entrance to the lesion using 3 to 8 mL of lidocaine mixed with 1:10^5^ diluted epinephrine. The Mammotome Elite^®^ needle with a bladed tip was directly inserted either through or just subjacent to the lesion. For the first pass, the cutter automatically rotated to sample both the target lesion and adjacent tissues. For the next pass, we rotated the needle shaft based on the relationship between the needle and lesion position. The needle was usually rotated from the 9- to 3-o’clock position until adequate tissue was collected in the collection cup. The procedure was completed after specimen mammography when the target showed calcifications on mammography. The new needle was used for each biopsy when there were more than two suspicious mass in a patient Manual compression was applied for 5 to 10 min to ensure hemostasis. Details of the biopsy procedure, including the reasons for use of this device instead of an automated gun, technical difficulties, core number, and complications were recorded. Bleeding longer than 20 min after the biopsy was considered as a complication. Severe pain terminating the procedure was also considered as a complication.

On the basis of the imaging findings, a metal clip was placed at the discretion of the radiologist performing the procedure. Criteria for clip deployment were that the postbiopsy ultrasound images demonstrate poor visualization of the target lesion due to removal. When requested by the surgeon, preoperative localization using intra-lesional carbon marking or wire insertion was performed.

Surgery was performed when the pathology revealed a malignancy, such as ductal carcinoma *in situ* (DCIS), invasive ductal carcinoma (IDC), or other invasive carcinoma. Patients with high-risk lesions, such as atypical ductal hyperplasia (ADH) or lobular carcinoma *in situ* (LCIS), were recommended to undergo surgical excision. For benign lesions with concordant imaging findings, the radiologist recommended US follow-up at 6, 12, and 24 months and mammographic follow-up at 12 and 24 months. If the lesions were highly suspicious despite benign pathologic results, surgical excision was recommended.

### Data collection

Data relating to the biopsy procedure; core numbers, calcification retrieval and complications, the clinical information about whether the procedure was rebiopsy and whether the lesion was initially palpable, imaging findings, and Wi-UVAB and surgical pathological results were recorded. The reason to choose a Wi-UVAB device for biopsy was also recorded based on the original report. Imaging variables derived from the report were the maximum size of the lesion on US, which was identifiable, and the lesion type on mammography and US, and the Breast Imaging Reporting and Data System (BI-RADS) assessment category integrated with US and mammography. Calcification retrieval was considered satisfactory if four or more than four calcific particles were obtained.

We investigated the yield of Wi-UVAB along the lesion type on US and BI-RADS category on US. We compared the Wi-UVAB and final diagnosis with combination from the surgical pathologic (n = 81) and follow-up US findings obtained more than 6-month post-biopsy (n = 22). The underestimation rate was also analyzed. The DCIS underestimation rate was the percentage of the number of lesions surgically proven as IDC or microinvasive carcinoma among the lesions operated after the diagnosis of DCIS at biopsy. High-risk lesion underestimation rate was the percentage of the number of lesions surgically proven as any carcinoma (IDC, DCIS, or microinvasive carcinoma) among the lesions operated after the diagnosis of ADH or LCIS at biopsy. False-negative cases were defined as all breast cancers (invasive cancer and DCIS) with a benign diagnosis at biopsy. Discordant cases were defined as the lesions assessed as discordant among those with benign Wi-UVAB results. The imaging and clinical characteristics relating to the underestimation rates were evaluated using the Fisher’s exact test in categorical data and Student’s t-test in nonparametric data. Values of p < 0.05 were considered to indicate a statistically significant difference.

Statistical analyses were performed by using software (SPSS version 16.0; SPSS, Chicago, Ill and MedCalc; MedCalc, Mariakerke, Belgium).

## Results

### Data relating to the biopsy procedure

In 103 cases of 96 women, four or more cores were usually obtained (range, 4–25; average, 10). The number of biopsies performed per lesion was 4–25, with a mean of 10.

In 7 cases (6.8%), a biopsy was reattempted with a needle in situ after emptying and reinstallation of a collection cup, because specimen retrieval was unsatisfactory by visual inspection or specimen mammography. Eventually, successful specimen retrieval of an adequate amount was achieved in all cases. Mean core number obtained in these uneasy cases was 20, ranging from 18 to 25. Complications were recorded in 9 patients (9.4%). Bleeding was not controlled within 20 min of post-procedural compression. In 6 of these patients, bleeding during the procedure obscured the lesion. No further treatment was required beyond applying additional compression up to total 35 min. One of these patients complained of breast swelling during the subsequent 24 h but no specific treatment was instituted. No patient complained of severe pain. Also, none of patient experienced a vasovagal response or skin defects.

Specimen mammography was performed in 66 patients with mammographic calcifications and showed a satisfactory amount of calcifications in 56 cases. Among 10 cases (15.2%) with inadequate calcifications (three or fewer calcific particles), 7 were malignancies and 3 were benign. Eight cases (7 in malignancy, 1 in benign) underwent surgery. There was no underestimated case.

Metallic clips were inserted in 21 cases and preoperative localization using intra-lesional carbon marking or wire insertion were performed in 33 cases.

### Clinical information and imaging findings

The major reason for using the Wi-UVAB device rather than the Tru-Cut biopsy gun was because the lesions were visible and suspicious but not apparent on US (n = 64). Minor reasons were that the lesion had a thin and slender shape (n = 8), small size (<7 mm; n = 18), lesion location near the implant (n = 2) or the chest wall muscle (n = 5), and biopsy intended for rebiopsy due to discordant benign results of 14-gauge core needle biopsy (n = 6). Most of the target lesions were nonpalpable (n = 96, 93.2%). Two of 7 palpable lesions were referred for rebiopsy and the remaining palpable lesion exhibited diffuse heterogeneity and calcifications on US (BI-RADS 4C and BI-RADS 4B, respectively). Six of 7 palpable lesions were confirmed to be malignant.

The lesion size on US was 0.4 to 5.1 cm (mean, 1.8 cm). The lesion type on mammography was calcifications in 66 cases (64.1%), masses, asymmetry or architectural distortion in 22 (21.4%), and negative findings in 15 (14.6%). The lesion type on US was a non-mass lesion with calcifications in 51 cases (49.5%), a non-mass lesion without calcifications in 18 (17.5%), a mass with calcifications in 8 (7.8%), and a mass without calcifications in 26 (25.2%) (Table 1). In 7 of 66 calcification cases on mammography, calcifications were not visible on US. The non-mass lesions appeared as non-uniform heterogeneous areas in 54 cases and duct-type tubular or branching lesions in 15. The integrated BI-RADS final assessment category was BI-RADS 4 in 97 lesions and BI-RADS 5 in 6 lesions. None of the lesions was BI-RADS ≤3 (Table 1).

**Table 1:** US Findings and BI-RADS classification in 103 Target Lesions of Wireless US-guided Vacuum-Assisted Biopsy in 96 Women.

	n (%)	Cancer, n (%)	High risk, n (%)	Benign, n (%)
**US findings**				
Non-mass lesion with calcifications	51 (49.5%)	36 (54.5%)	4 (33.3%)	9 (36.0%)
Non-mass lesion without calcifications	18 (17.5%)	9 (13.6)	2 (16.7)	7 (28.0)
Mass with calcification	8 (7.8%)	5 (7.6)	4 (33.3)	3 (12.0)
Mass without calcifications	26 (25.2)	16 (24.2)	2 (16.7)	6 (24.0)
**BI-RADS classification**				
BI-RADS Category 4	97 (94.2)	62 (93.9)	10 (83.3)	25 (100)
4A	37 (35.9)	14 (21.2)	7(58.3)	16 (64.0)
4B	35 (34.0)	25 (37.9)	1 (8.3)	9 (36.0)
4C	25 (24.3)	23 (34.8)	2 (16.7)	0 (0.0)
BI-RADS Category 5	6 (5.8)	4 (6.1)	2 (16.7)	0 (0.0)
**Total**	103 (100.0)	66 (64.1)	12 (11.7)	25 (24.3)

### Pathological results

**Fig 1:**
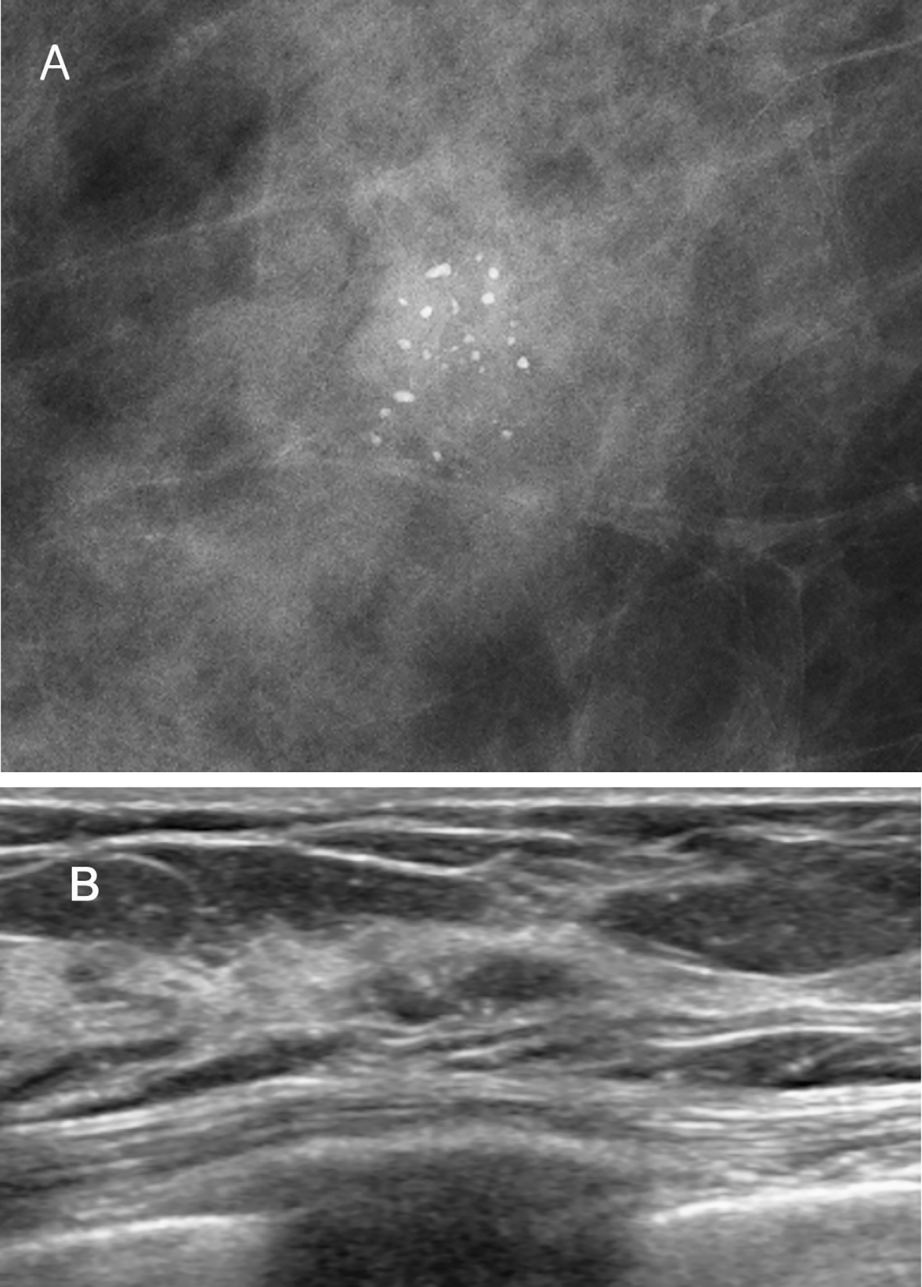
A 64-year-old asymptomatic female with an abnormality on screening mammography. (A) Magnification mammography demonstrates pleomorphic clustered microcalcifications in the right lower outer breast. (B) This lesion shows tubular isoechoic lesion with microcalcifications on US. The pathologic findings of both Wi-UVAB and surgical excision were pleomorphic LCIS.

The histopathological diagnoses of the biopsy and surgical specimens are shown in Table 2. The malignancy rate was 64.1%. There were no significant difference between the malignancy rate in the lesions with calcifications and the rate in the lesions without calcifications (62.1% vs. 37.9%, respectively; p = 0.45). Among the 97 BI-RADS 4 lesions, the malignancy rate was 63.9% (Table 1). Among the six BI-RADS 5 lesions, 4 were malignant and 2 lwere high risk lesions. One of the high risk lesions classified as BI-RADS 5 was a pleomorphic LCIS that appeared as a tubular isoechoic lesion with microcalcifications on US. In this case, there were grouped pleomorphic calcifications on mammography (Fig 1). Final surgical pathology revealed pleomorphic LCIS. The other was ADH that presented as a faint non-mass lesion with microcalcifications on US that were segmental and fine pleomorphic on mammography. Final surgical result was DCIS.

**Table 2:** Histologic Results of Wireless US-guided Vacuum-Assisted Biopsy (Wi-UVAB) and Surgical Biopsy in 103 Cases in 96 Women.

UVAB Histology (n = 103)	Surgical Histology (n = 81)		N/A
	Accurate diagnosis (n = 67)	Underestimation (n = 14)	
**Malignancy (n = 66)**			
IDC (18)	IDC (18)		-
MIBC (7)	MIBC (4), IDC (3)		-
DCIS (40)	DCIS (29)	MIBC (9), Invasive cancer (2)	-
ILC (1)	ILC (1)		-
**High-risk lesions (n = 12)**			
ADH or columnar cell change with atypia (7)	ADH (2)	DCIS (2)	Imaging follow up (3)
LCIS (3)	LCIS (2)	ILC (1)	-
Intraductal papilloma with atypia (2)	Intraductal papilloma with atypia (2)		-
**Benign lesions (n = 25)**			
Specific benign abnormality (12)	Lobulocentric granulomatous inflammation (1), Intraductal papilloma (1)		Imaging follow up (10)
Nonspecific benign abnormality (13)	Fibroadenoma(1), Nonspecific benign abnormality (3)		Imaging follow up (9)

Number in parentheses are the number of the cases. MIBC, microinvasive breast carcinoma; IDC, invasive ductal carcinoma; ILC, invasive lobular carcinoma; DCIS, ductal carcinoma in situ; LCIS, lobular carcinoma in situ; N/A, Not available due to non-operation.

There were 6 rebiopsy cases with imaging-pathology discordance at initial Tru-cut core needle biopsy. Among them, 4 cases classified as BI-RADS 4B (n = 1) or 4C (n = 3) were upgraded to malignancy after rebiopsy. The other 2 BI-RADS 4A cases revealed stromal fibrosis.

All 66 malignant lesions underwent surgical biopsy; 11 of the 40 DCIS lesions revealed discordant final results (microinvasive ductal carcinoma, n = 9; invasive cancer, n = 2) (Table 2). Twelve lesions were high-risk lesions (ADH, n = 7; LCIS, n = 3; intraductal papilloma with atypia, n = 2). The 3 LCIS lesions were finally confirmed to be 2 LCIS and 1 ILC after the surgery.

Among 7 ADH lesions, 2 proved to be DCIS (Table 2). The 3 patients with ADH lesions refused surgery and have been followed up with US and mammography (15 months and 19 months, respectively). These imaging findings demonstrated stability after 12 months.

Twenty-five lesions were benign (specific benign histology, n = 12; nonspecific benign histology, n = 13) (Table 2). The lesions with a specific benign histology included intraductal papilloma (n = 1), sclerosing adenosis (n = 4), fibroadenoma (n = 1), lobulocentric granulomatous inflammation (n = 1), diabetic mastopathy (n = 1), and columnar cell change (n = 4). The lesions with nonspecific benign histology included stromal fibrosis (n = 6) and fibrocystic change (n = 7). No repeat biopsy was required because of imaging and pathologic concordance. Follow-up imaging at >6 months (range, 6–18 months; mean, 10.1 months) showed no late development of malignancy in all benign lesions. There were neither false-negative nor discordant cases that required additional diagnostic procedures in the cases with benign Wi-UVAB histology.

DCIS underestimation rate was 27.5% (11/40). Two DCIS lesions were upgraded to IDC; 1 was a 0.90-cm IDC and the other was a 0.14-cm mucinous carcinoma. Nine DCIS lesions upgraded to microinvasive ductal carcinoma. Most of the underestimated lesions (8/11) showed non-mass lesions with calcifications on US.

**Fig 2:**
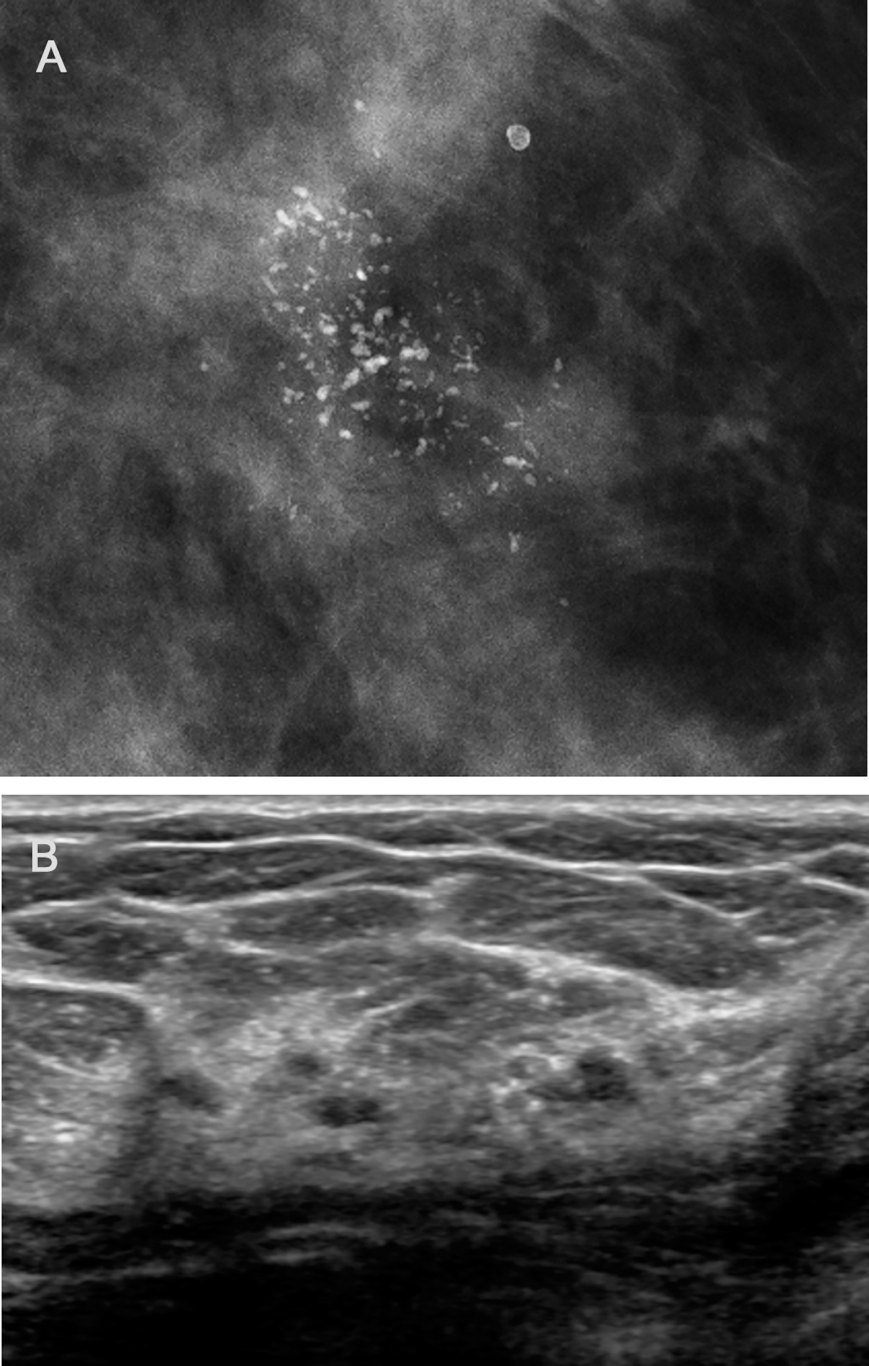
A 48 year-old asymptomatic female with an abnormality on screening mammography. (A) Magnification mammography shows pleomorphic clustered microcalcifications in the left upper outer breast. (B) On US, corresponding lesion presents multiple echogenic dots indicating micocalcifications. The pathologic results of Wi-UVAB were LCIS, but ILC was found at following surgery.

High risk lesion underestimation rate was 33.3% (3/9). One LCIS lesion proved to be ILC of 0.6 cm in 3.5 cm-sized LCIS (Fig 2), and 2 ADH cases revealed an 8-cm DCIS and a 0.5-cm DCIS, respectively. All high risk underestimated lesions revealed segmental or grouped pleomorphic calcification on mammography and non-mass lesion with calcifications on US.

Table 3 compares the accurate diagnoses (n = 67) and underestimations (n = 14) according to patient and lesion variables. No difference was found between the accurate diagnosis and underestimation groups in terms of age (p = 0.50), lesion size (p = 0.48), lesion type on US (p = 0.16), BI-RADS category (p = 0.28), number of core specimens (p = 0.23), or the number of calcification (p = 0.17).

**Table 3:** Comparison of Accurately Diagnosed and Underestimated Lesions.

Feature	Accurately Diagnosed (n = 67)	Underestimated (n = 14)	*p*
**Mean ± SD patient age (year)**	50.3 ± 8.9	54.5 ± 10.1	0.50
**Mean ± SD lesion size (cm)**	1.8 ± 1.2	2.3 ± 1.4	0.48
**Lesion type on US**			0.16
Non-mass lesion with calcifications	33	10	
Non-mass lesion without calcifications	12	1	
Mass with calcification	4	2	
Mass without calcifications	18	1	
**BI-RADS category**			0.28
4	63	12	
5	4	2	
**No. of specimen**	9.9 ± 4.6	9.9 ± 3.6	0.23
≤ 5	11	0	
6–10	31	7	
≥ 11	25	7	
**No. of calcification**			0.17
≥ 4	29	12	
< 4	8	0	

P values were calculated by student T-test and fisher’s exact test.

## Discussion

Percutaneous breast biopsies have transitioned from fine-needle aspiration biopsy to VAB, allowing for more accurate diagnosis of suspicious breast lesions and decreasing insufficient or inconclusive diagnoses. To obtain adequate tissue samples, Tru-Cut biopsy often requires repeated insertion and reinsertion of the needles through the lesion, causing patient discomfort and increasing the risk of complications [5].

Vacuum-assisted devices allow for single insertion with multiple contiguous samples to reduce non-diagnostic or ambiguous results and patient discomfort. However, it is quite bothersome for physicians to prepare and setup the large equipment of conventional VAB. A noteworthy advantage is that Wi-UVAB does not require additional time to prepare and setup the machine, allowing for biopsy to be promptly performed without delay. Wi-UVAB may offer easy scheduling with diagnostic US at the same day, differently from the conventional VAB. Also comparing with Tru-cut biopsy, Wi-UVAB does not require the time for in-and-out workout of the needle between samples, which can shorten the procedure time.

The reported complication rate associated with conventional vacuum-assisted devices ranges from 0% to 9%, and the most serious adverse events reported include severe bleeding and skin defects [6–9]. A recent study reported virtually no complications using the Wi-UVAB [5]. The present study also showed minimal complications; moderate amount of bleeding in 9 patients (9.4%) that lasted beyond the standard 10 min of postbiopsy manual compression. One of these patients complained of breast swelling during the subsequent 24 h but no specific treatment was instituted.

In a published study on the use of Wi-UVAB, most lesions exhibited opacities on mammography (70.6%), palpable nodes on US (74.6%), or a suspicious BI-RADS category (57.4%) [5]. In our study, most of the lesions were nonpalpable (93.2%), non-mass lesions with calcifications on US (49.5%), and all had a suspicious BI-RADS category (100.0%). In such case, VAB would be used rather than Tru-cut biopsy because an acquisition of representative samples by Tru-cut core needle biopsy is difficult as the lesions were broad with indistinct margins. US-guided biopsy may be preferable than stereotactic biopsy in terms of real-time, patient comfort, radiation exposure, procedure time, and cost effectiveness. Our study demonstrates that the Wi-UVAB device could be the device of choice for non-mass lesions because it allows fast acquisition of a large tissue volume resulting in high diagnostic accuracy of malignancy and does not necessarily require precise localization of the needle.

Tru-Cut core needle biopsy reportedly has a false-negative rate of 0% [10–14]to 9%[15, 16]. Most studies with a false negative rate of 0% had small populations. [10–12, 14] The absence of a false negative case in our study may also have resulted result from the small number of benign cases. However, considering that four rebiopy cases diagnosed benign lesion at core needle biopsy turned out to be malignant at Wi-UVAB, we can assume that the false-negative rate of Wi-UVAB is lower than that of Tru-cut biopsy due to increase the volume of the sample obtained.

Presently, the high risk lesion underestimation rate was 33.3%, which is almost identical to the 31–33% reported in other Tru-cut biopsy studies.[17, 18] The high risk breast lesions in our study were ADH, LCIS, and intraductal papilloma with atypia. These lesions are an indication for surgical excision because of a risk for malignancy.

ADH is a pathologic entity that has some, but not all features, of DCIS. Thus, sampling error can result in underestimation.[19] In one study, the ADH underestimation rate was 18.8% as the complete removal of the lesion with the VAB.^19^ When the specimens were only partially collected from the lesions, the underestimation was 31.3%. In our study, half of ADH which underwent surgical excision upgraded to DCIS. Our high underestimation rate may be attributed to smaller size of needle (13-gauge) than conventional VABB (11-gauge), resulting in partial removal of the target lesions. However, the identification of ADH in a core biopsy specimen is clinically important because of the high prevalence of DCIS or invasive ductal carcinoma associated with this abnormality in as many as 56% of cases.[20, 21]

The present DCIS underestimation rate of 27.5% is lower than reported underestimation rate of Tru-Cut biopsy (39%-67%)[6, 14, 22–24] and is similar with reported underestimation rate of VAB.(17–41%).[22, 23] The lower DCIS underestimation rate with larger tissue acquisition devices is attributed to the larger volume of tissue acquired, with a decrease in sampling error. However, our DCIS underestimation lesions showed relative high proportion of microinvasive cancer (9/11). Underestimation rate for calcifications was reported to be significantly higher than that for masses.^3^ This difference might have resulted in a slightly higher proportion of microinvasive cancer in our study, because most of target lesions had visible but unapparent findings at the area of calcifications on US.

To identify possible factors involved in invasiveness, we compared the underestimated and accurate diagnosed groups; no statistically significant differences were evident between the groups with regard to clinical and imaging characteristics.

Underestimation has been reported as being more frequent when 10 or fewer specimens were obtained.[25] However, the core number was not statistically different between the underestimated and accurately diagnosed groups (p = 0.23) in our study. The mean core number of reattempted biopsy cases was two times higher than that of other cases. We assume that the number of core obtained may not reflect the degree of thoroughness of sampling; instead, a greater number of cores may indicate an initial failure to retrieve sufficient samples.

Another recent study showed that DCIS underestimation rate was significantly related to presence of palpability, mass and calcification by US and biopsy method.[26] Lesions >20 mm in size have been associated with invasive components on final pathology. [27] However, in our study, there was no significant difference between underestimated and accurate diagnosed group in terms of lesion size, retrieval of calcification, and ultrasonographic features of lesions, such as mass or non-mass.

Our study had several limitations. First, the sample size of benign and high risk lesion was relatively small. False negative rate and high risk underestimation rate may influenced by the sample size. Second, there were cases that did not undergo surgical excision to confirm the final pathology, even though the lesions showed stability on US follow-up (range, 6–20; mean 11.1months). A change could be possible afterwards. Third, in our study, only experts carried out the biopsies. If physicians lack experience with Wi-UVAB, training is required to obtain acceptable false negative and underestimation rates with relatively few complications from the procedure.

In conclusion, we believe that this Wi-UVAB device is safe and accurate. It is useful when US-guided biopsies are preferred and when suspicious nonpalpable, non-mass lesions exhibit visible but unapparent calcifications on US. Although the histological results of DCIS may be upgraded to subcentimeter invasive cancer or microinvasive ductal carcinoma and high risk lesion may turn out to be malignancy, false-negative biopsy results can be avoided by use of Wi-UVAB.
